# Pregnancy-related conditions and premature coronary heart disease in adult offspring

**DOI:** 10.1136/heartasia-2017-010896

**Published:** 2017-05-24

**Authors:** Andriany Qanitha, Bastianus A J M de Mol, David P Burgner, Peter Kabo, Dara R Pabittei, Irawan Yusuf, Cuno S P M Uiterwaal

**Affiliations:** 1 Department of Cardio-thoracic Surgery, AMC Heart Center, Academic Medical Center, University of Amsterdam, Amsterdam, The Netherlands; 2 Department of Physiology, Faculty of Medicine, University of Hasanuddin, Makassar, Indonesia; 3 Murdoch Childrens Research Institute, Parkville, Victoria, Australia; 4 Department of Paediatrics, University of Melbourne, Parkville, Victoria, Australia; 5 Department of Paediatrics, Monash University, Clayton, Victoria, Australia; 6 Department of Cardiology and Vascular Medicine, Faculty of Medicine, University of Hasanuddin, Makassar, Indonesia; 7 Julius Center for Health Sciences and Primary Care, University Medical Center Utrecht, Utrecht, The Netherlands

**Keywords:** INFECTION

## Abstract

**Objective:**

To investigate the association between complications during pregnancy and premature coronary heart disease in adult offspring.

**Methods:**

We conducted a population-based case-control study of 153 Indonesian patients with a first acute coronary syndrome (ACS) (age ≤55 years) and 153 age-matched and sex-matched controls. Data on complications during pregnancy (high blood pressure, preterm delivery) and maternal infections in pregnancy were obtained, together with sociodemographic data, clinical profiles, laboratory measurements and adulthood cardiovascular disease (CVD) risk factors at hospital admission or enrolment. Conditional logistic regression was performed to assess the association between overall pregnancy complications, and specific groupings of complications and premature ACS.

**Results:**

Pregnancy-related hypertension and infection were more common in mothers of cases than controls. Pregnancy complications were associated with premature offspring ACS (OR 2.9, 95% CI 1.4 to 6.0, p=0.004), and the association persisted in fully adjusted analyses (OR_adjusted_ 4.5, 1.1 to 18.1, p=0.036). In subgroup analyses, pregnancy-related high blood pressure (OR_adjusted_ 5.0, 1.0 to 24.7, p=0.050) and maternal infections (OR_adjusted_ 5.2, 1.1 to 24.2, p=0.035) were associated with offspring ACS.

**Conclusions:**

Offspring of mothers with complications during pregnancy have an increased risk for premature ACS in adulthood, which may be of particular relevance in populations in transition, where the incidence of both pregnancy-related morbidity and CVD are high.

## Introduction

Increasing evidence indicates that in utero exposures contribute to adult disease, in addition to childhood and adulthood exposures.^1^ In this light, numerous studies and meta-analyses have been conducted on maternal pregnancy complications and their relationship to adult cardiovascular disease (CVD) risk factors in the offspring. The associations between gestational hypertension,^2^ ^3^ pre-eclampsia^3–5^ and preterm birth,^6^ with adult obesity, glucose intolerance, hypertension and metabolic syndrome in the offspring are widely accepted. Although the underlying mechanisms are poorly understood, a range of disruptions in fetal growth and unfavourable intrauterine environment—to which a variety of pregnancy-related complications may contribute—might act through common pathways,^7^ leading to early metabolic^8^ and arterial vasculature changes^9^ that may initiate earlier and accelerated atherosclerosis.^9^ Recent studies show that offspring of mothers with complications during pregnancy have increased CVD risk factors in childhood and adolescence.^4^ ^5^ ^10^ However, it is uncertain if these pregnancy complications are related with the development of clinically manifest CVD, particularly in young adult offspring, or whether specific complications have more marked effects.

WHO has reported that 99% of all global maternal deaths due to pregnancy complications occur in the low-income and middle-income countries.^11^ In contrast to Western populations, the major causes of maternal pregnancy complications in these regions are unsafe abortion (8%), infections (11%), high blood pressure (14%), severe bleeding (27%) and pre-existing conditions (28%).^11^ In addition, approximately 80% of global CVD deaths occur in low-income and middle-income countries,^12^ and an estimated half of the global CVD burden occurs in Asia.^13^ South-East Asia has a population of over 600 million—the majority younger than 65 years—and has faced a rapid epidemiological transition.^14^ Compared with the high-income countries, South-East Asian countries have a higher prevalence of cardiovascular risk factors in young adults^15^ that is reflected in high rates of premature deaths (age <60 years^15^) due to non-communicable diseases (NCDs), primarily CVD.^14^ ^15^ Of the 7.9 million annual NCD deaths in this region, 34% occurred before the age of 60 years compared with 16% in the European region and 23% in the rest of the world.^15^ A striking observation is that deaths from CVD occur 5–10 years earlier in Asia compared with Western countries.^16^ Both maternal pregnancy complications and premature CVD are major health issues, particularly in South-East Asia.

We hypothesised that complications in pregnancy may contribute to the increased incidence of premature CVD in this setting. Therefore, we conducted a population-based case-control study of a first acute coronary syndrome (ACS) episode in Indonesia to investigate the association between complicated pregnancy and the occurrence of early coronary heart disease (CHD) in adult offspring.

## Methods

This study was conducted between February 2013 and December 2014 in Makassar Cardiac Center, Wahidin Sudirohusodo Hospital, the general academic and referral hospital in East Indonesia.

### Cases

Cases were defined as patients with a first ACS event occurring at age ≤55 years. After obtaining written informed consent, we enrolled 153 consecutive new patients, who were admitted to the cardiovascular care unit with ACS, defined as unstable angina (UA), non-ST segment elevation myocardial infarction (NSTEMI) and ST segment elevation myocardial infarction (STEMI).^17^

Cardiologists made a diagnosis of ACS in patients with acute cardiac chest pain based on clinical presentations, ECG, and elevated biomarker of cardiomyocyte injury (cardiac troponin (cTn) and/or creatine kinase muscle and brain (CKMB)). Patients were diagnosed with STEMI if they had new or presumed new significant ST segment T wave (ST-T) changes or new left bundle branch block, and/or development of pathological Q waves on ECG, with an increase of cardiac troponin T (cTn-T) and/or CKMB, at least one value above the 99th centile of a normal reference population (upper reference limit).^18^ ^19^ NSTEMI diagnosis was defined if clinical presentation is compatible with myocardial ischaemia, followed by an increased cTnT and/or CKMB, without new ST segment elevation on the presentation or subsequent ECG. UA was defined as ischaemic chest pain at rest or minimal exertion persisting for >20 min in the absence of cardiomyocyte necrosis.^18^ Patients were excluded if they had a history of previous CVD, or showed no evidence of significant atherosclerosis on coronary angiography.

### Controls

We enrolled young adult controls from the general population in equal numbers to cases. We matched the cases and controls for age (±3 years) and sex. Controls were sampled from the same residential neighbourhood as the cases and were invited to visit a primary healthcare centre for clinical examination, laboratory measurements and a questionnaire interview. To ensure incidence-density sampling, a control was recruited in the same week as a case was enrolled. Controls were excluded if they had any previous diagnosis of CVD, had current clinical features of a cardiovascular event or were unable to give written informed consent.

### Data collection

A detailed questionnaire was administered to all cases and controls. In the cases group, we obtained baseline data from medical records, physical examination and interview, including age, sex, occupation, monthly income, education level, dietary pattern, physical activity, smoking status, parental history of CVD, as well as previous history of hypertension and diabetes (detailed methods were presented as online supplementary material). Plasma glucose, lipid profiles, uric acid, renal and liver function were measured following a minimum 8 hours fast in hospital laboratories using standard techniques within 24 hours of hospital admission for all cases. We collected identical data from controls at their primary healthcare centre visit.

10.1136/heartasia-2017-010896.supp1 Supplementary data

### Pregnancy complications

Exposure to pregnancy complication was considered positive if the mothers of participants reported or had been diagnosed with significant predefined complications during the pregnancy with the case or control. We included the common pregnancy complications in the South-East Asian region as follows: high blood pressure, preterm delivery and maternal infections during pregnancy. For maternal infections, we classified upper respiratory infection, lower respiratory infection, gastrointestinal infection (including typhoid fever), genitourinary tract infections, dengue fever, malaria, varicella, measles and unspecified infections (defined as fever of unknown cause for ≥3 days or requiring hospitalisation)). Reliable medical records in hospital and primary healthcare in Indonesia did not exist at the time of pregnancy. Therefore data on pregnancy complications were obtained using take-home questionnaires filled by participants (cases and controls), enabling information to be sourced from their mothers or other family members who were present throughout the pregnancy. We collected the questionnaire by visiting participants' houses. We subsequently verified the data by a further interview to minimise missing data and resolve ambiguities.

### Confounding variables and regression models

There are several possible confounders that may be relevant to analysis of an association between pregnancy complications and premature ACS in adult offspring. The following variables were therefore included in the regression analyses: adulthood lifestyle (current smoking status); dietary pattern (high salty food and monosodium glutamate (MSG) intake, and high fatty food intake); socioeconomic status (monthly income and college education); parental history of CVD; childhood infections,^20^ ^21^ and adulthood cardiovascular risk factors (hypertension, raised fasting plasma glucose (≥6.1 mmol/L), low-density lipoprotein (LDL)-cholesterol, high-density lipoprotein (HDL)-cholesterol and body mass index (BMI)).

In the present study, we included childhood infections (yes/no) as a confounding variable, as we^20^ and others^21^ have reported that childhood infection is independently associated with an early CVD hospitalisation in adult life. We defined exposure to childhood infections if participants had experienced at least one severe infection during early childhood (0–5 years of age), as previously reported.^20^

The selection of confounders in this study is crucial. As pregnancy complication is a broad categorisation encompassing various conditions during pregnancy, there are several extraneous factors that potentially act either as a confounding or as an intermediate variable. Maternal infections during pregnancy may be related with offspring childhood infections, while maternal high blood pressure in pregnancy is related with offspring hypertension in adult life.^3^ There is considerable evidence of association between pregnancy complications and the development of cardiovascular risk factors in adulthood.^5^ ^6^ For that reason, we assumed that several adulthood cardiovascular risk factors (eg, hypertension, diabetes and metabolic syndrome) plausibly are intermediate variables that may affect the true association between maternal pregnancy complications and premature CVD in the offspring and hence lie on the causal pathway between pregnancy complications and adult CVD. Therefore to investigate whether these confounding and/or intermediate variables affected the associations, we performed stepwise analyses in four regression models as follows:

- Crude=Univariable (matched for age and sex)
- Model 1=Crude+adulthood lifestyle+dietary pattern +socioeconomic status
- Model 2=Model 1+parental history of CVD
- Model 3=Model 2+childhood infections
- Model 4=Model 3+adulthood cardiovascular risk factors

### Data analysis

Baseline characteristics of cases and controls were first tabulated for descriptive purposes and confounder assessment. To assess group differences, paired t-tests were performed with normal distributions and non-parametrical Wilcoxon signed-rank tests for skewed distributions. For categorical variables, McNemar's χ^2^ tests were applied. We reported median and IQR for skewed data. Main results were expressed as crude and adjusted ORs from univariable and multivariable conditional logistic regressions, with their 95% CI, corresponding to two-sided p values <0.05.

We initially performed the overall analyses dichotomising the presence of pregnancy complications as the exposure. We then performed complication-specific analyses for two subgroups: (1) high blood pressure, and (2) maternal infections. We did not apply further subgroup analyses for preterm delivery as this complication showed very low numbers among cases and controls. In subgroup analyses, we restricted the analyses to the subset group of interest as ‘exposed’ group, while other pregnancy complications and those without any pregnancy complications were classified as ‘unexposed’ group. Statistical analyses were performed with IBM SPSS V.23.0.

## Results

Baseline characteristics of cases and controls are shown in table 1. The mean (SD) age was 47 (6.3) years (range 28–55 years) and 81.7% were male. Compared with controls, cases were more likely to have a higher fasting plasma glucose, a lower HDL-cholesterol level, a lower LDL-cholesterol level, metabolic syndrome, to report hypertension and/or diabetes, to have had college education, to be a current smoker, to consume more salty food and MSG, and more fatty food. Cases and controls had approximately equal monthly income and similar BMI (table 1).

**Table 1:** Baseline characteristics of cases and controls (adult offspring)†

Variables	Case (n=153)	Control (n=153)	p Value
Male (%)	125 (81.7)	125 (81.7)	1.000
Age (years)	47.1±6.2	46.9±6.4	0.121
Systolic BP (mm Hg)	120.7±21.2	120.3±21.6	0.867
Diastolic BP (mm Hg)	78.2±13.7	80.2±14.1)	0.221
Plasma glucose (mmol/L)‡	6.9 (5.7–9.6)	4.8 (4.3–5.6)	<0.001*
Raised plasma glucose§ (%)	103 (67.3)	25 (16.3)	<0.001*
Total cholesterol (mmol/L)‡	5.1 (4.4–5.9)	5.3 (4.8–5.9)	0.541
Triglycerides (mmol/L)‡	1.6 (1.1–2.4)	1.5 (1.2–2.3)	0.287
HDL-chol (mmol/L)	0.9±0.2	1.2±0.3	<0.001*
LDL-chol (mmol/L)	3.5±1.3	3.9±0.9	0.002*
Body mass index (kg/m^2^)	24.4±3.1	24.4±4.1	0.962
Waist circumference (cm)	86.3±7.8	88.7±10.2	0.013*
Metabolic syndrome (%)	92 (60.1)	30 (19.6)	<0.001*
History of hypertension (%)	99 (64.7)	60 (39.2)	<0.001*
History of diabetes mellitus (%)	44 (28.8)	17 (11.1)	<0.001*
Maternal history of CVD¶ (%)	21 (13.7)	27 (17.6)	0.430
Paternal history of CVD** (%)	22 (14.4)	25 (16.3)	0.755
Monthly income (IDR 1,810,000)†† (%)	82 (53.6)	68 (44.4)	0.146
College education (%)	67 (43.8)	48 (31.4)	0.034*
High salty food/MSG intake (%)	73 (47.7)	47 (30.7)	0.002*
High fatty food intake (%)	31 (20.3)	9 (5.9)	<0.001*
Less fibre (%)	9 (5.9)	11 (7.2)	0.824
Current smoker (%)	70 (45.8)	49 (32.0)	0.015*
Former smoker (%)	36 (23.5)	46 (30.1)	0.203
Physical inactivity (%)	101 (66.0)	117 (76.5)	0.060

*p<0.05.

†Values are n (%) or means±SD, unless otherwise stated. Comparison of baseline characteristics between cases and controls (adult offspring) was performed using paired-samples t-test for continuous variables and McNemar's χ^2^ test for categorical variables.

‡Values are medians (Q1–Q3). Comparison was performed using Wilcoxon sign-rank test for paired samples.

§Defined as fasting plasma glucose ≥6.1 mmol/L.

¶Maternal history of CVD defined as mother had CVD at age <65 years.

**Paternal history of CVD defined as father had CVD at age <55 years.

††The cut-off point based on the national average of minimum wages for decent living in Indonesia in 2015.

BP, blood pressure; CVD, cardiovascular disease; HDL-chol, high density lipoprotein-cholesterol; IDR, Indonesian Rupiah; LDL-chol, low density lipoprotein-cholesterol; MSG, monosodium glutamate.

Table 2 lists the details of pregnancy complications of the participants. Thirty (19.6%) mothers of cases had a complication during pregnancy, compared with 11 (7.2%) mothers of controls (p=0.003).

**Table 2:** Pregnancy-related conditions sustained by mothers of cases and controls†

Pregnancy complications	Cases (n=153)	Controls (n=153)	p Value
Maternal high blood pressure	9 (5.9)	3 (2.0)	0.146
Preterm delivery‡	1 (0.7)	1 (0.7)	1.0
Maternal infections	20 (13.1)	7 (4.6)	0.015*
Upper respiratory infection§	5 (3.3)	2 (1.3)	0.453
Lower respiratory infection¶	2 (1.3)	0 (0.0)	0.157
Gastrointestinal infection**	4 (2.6)	0 (0.0)	0.046*
Unspecified infections††	9 (5.9)	5 (3.3)	0.388
Total complications	30 (19.6)	11 (7.2)	0.003*

*p<0.005.

†Values are n (%). Comparison was made using exact McNemar's χ^2^ test.

‡Defined as babies born alive before 37 weeks of gestational age.^6^

§Consisted of influenza, tonsillitis, pharyngitis, laryngitis, sinusitis or otitis media.

¶Consisted of bronchitis, pneumonia and tuberculosis.

**Consisted of diarrhoea and typhoid fever.

††Defined as fever of unknown cause for ≥3 days or requiring hospitalisation.

Table 3 shows the results of univariable and multivariable regressions as the main analyses. In univariable analysis, participants whose mothers had a pregnancy complication had an almost threefold increased odds of acquiring premature ACS in adulthood compared with those whose mothers had no complication. For overall pregnancy complications, these associations remained statistically significant in multivariable analyses (OR_adjusted_ 4.5, 95% CI 1.1 to 18.1, p=0.036). In subgroup analyses, maternal high blood pressure (OR_adjusted_ 5.0, 1.0 to 24.7, p=0.050) and maternal infections (OR_adjusted_ 5.2, 1.1 to 24.2, p=0.035) during pregnancy were related with offspring premature ACS.

**Table 3:** ORs for maternal pregnancy complications†

Pregnancy complications	OR (95% CI)	p Value
Overall pregnancy complications		
Crude‡	2.90 (1.41 to 5.95)	0.004*
Model 1§	3.16 (1.42 to 7.08)	0.005*
Model 2¶	4.06 (1.68 to 9.79)	0.002*
Model 3**	3.25 (1.27 to 8.28)	0.014*
Model 4††	4.47 (1.10 to 18.13)	0.036*
Maternal high blood pressure		
Crude‡	3.00 (0.81 to 11.08)	0.099
Model 1§	5.18 (1.02 to 26.33)	0.048*
Model 2¶	4.80 (1.03 to 22.48)	0.046*
Model 3**	4.83 (0.81 to 28.68)‡‡	0.083‡‡
Model 4§§	4.96 (1.00 to 24.66)	0.050*
Maternal infections		
Crude‡	3.17 (1.27 to 7.93)	0.014*
Model 1§	3.00 (1.14 to 7.90)	0.026*
Model 2¶	3.66 (1.30 to 10.26)	0.014*
Model 3**	2.81 (0.94 to 8.37)‡‡	0.064‡‡
Model 4††	5.21 (1.13 to 24.20)	0.035*

*p<0.05.

†Univariable and multivariable analyses were done by conditional logistic regression for matched data.

‡Crude was univariable analysis.

§Model 1 was adjusted for current smoking status, high salty food and MSG intake, high fatty food intake, monthly income and college education.

¶Model 2 was Model 1 plus adjusted for parental history of CVD.

**Model 3 was Model 2 plus adjusted for childhood infections.

††Model 4 was Model 3 plus adjusted for adulthood cardiovascular risk factors (hypertension, raised fasting plasma glucose, LDL-cholesterol, HDL-cholesterol and BMI).

‡‡Multicollinearity was assumed.

§§Model 4 was not adjusted for offspring adulthood hypertension and childhood infections because of strong collinearity among these variables.

BMI, body mass index; CVD, cardiovascular disease; HDL, high-density lipoprotein; LDL, low-density lipoprotein; MSG, monosodium glutamate.

## Discussion

In this case-control study of Indonesian adults, complications during pregnancy were associated with premature ACS in adult offspring. These associations were independent of adulthood cardiovascular risk factors, dietary pattern, socioeconomic status, parental history of CVD and childhood infections. In subgroup analyses, those born following pregnancies complicated by infection were significantly at increased risk of premature ACS. To the best of our knowledge, this is the first report of an association between maternal infections in pregnancy and CVD in the offspring.

Although CVD risk factors burden has transited from rich to poor populations in Indonesia,^22^ this risk burden is still more marked in the rich people. Apparently, in our study population, the cases—of which most of them came from middle to high socioeconomic level—had more traditional CVD risk factors, had higher education attainment, were often smokers, and consumed more salty/MSG and fatty food compared with controls.

It is generally accepted that maternal problems such as hypertension and infections during pregnancy, and preterm delivery may directly affect birth outcomes in the newborns. There is also increasing evidence relating these exposures to later outcomes in childhood^2^ ^4^ ^10^ and adulthood.^6^ Several studies have shown that offspring from pregnancies complicated by pre-eclampsia or gestational hypertension have higher blood pressure, BMI and cholesterol during childhood^10^ and adolescence.^2^ ^4^ Furthermore, in a recent meta-analysis, preterm delivery was associated with higher blood pressure and increased plasma LDL in adulthood.^6^ Delivery prior to 34 weeks gestational age has also been associated with all-cause mortality between 15 years and 45 years of age in adult offspring.^23^

This is the first study to address the relationship between maternal infections during pregnancy and clinical CVD in adult offspring. The mechanisms are unclear, as the absolute number of mothers reporting infection in pregnancy is modest, the diagnoses were by maternal recall, and antibiotic and other treatments are largely unknown. Further longitudinal studies are warranted to confirm this finding, interrogate mechanisms and provide a platform for translation. Previous studies and meta-analyses on pregnancies complicated by maternal infection have reported increased risk of adverse neurodevelopmental outcomes in the offspring, including autism spectrum disorder,^24^ schizophrenia^25^ and brain damage.^26^ Another study reported the increased risk of congenital heart defects in the newborns after pregnancy complicated by maternal febrile illness.^27^

Mounting evidence suggests that impaired fetal growth is associated with increased risk of adult CHD.^9^ Although the exact mechanisms are unknown, pregnancy-related morbidity may induce an adverse intrauterine environment and compromise fetal nutrition,^28^ consequently impairing the fetal growth. During fetal development, a range of disruptions in pregnancy may act through common pathways,^7^ induced early metabolic^8^ and arterial^9^ changes, and programme a predisposition to atherosclerosis.^9^ Impaired in utero growth is associated with changes to both arterial function (endothelial dysfunction) and structure (increased wall thickness).^9^ This is in keeping with animal models, which report that exposure to cardiovascular risk factors such as hypercholesterolaemia during pregnancy may contribute to later atherosclerosis development.^29^ These recent findings indicate that intrauterine factors, in tandem with genetic predisposition and postnatal environmental exposures might contribute to a precocious atherosclerosis,^9^ and increase the risk of later CVD.

The longitudinal Helsinki Birth Cohort Study reported a relationship between pre-eclampsia and pregnancy hypertension with the occurrence of stroke in adult offspring, but not with CHD.^30^ However our study is among the first to investigate the association between the common complications of pregnancy with premature CHD in adult offspring in a low-income and middle-income population in South-East Asia, where differences in both heritable and environmental factors may lead to divergent causal pathways. Our study adds to the existing knowledge on maternal pregnancy complications and cardiovascular risk factors, notably that such exposures may partly contribute to premature CVD in these settings.

Interpretation of these novel but preliminary data should be considered within the limitations of longitudinal data in this setting. The potential mechanisms that underlie the association between pregnancy complications and premature ACS in adult offspring may involve fetal growth impairment,^8^ ^9^ ^31^ for which birth weight is a proxy. Unfortunately, we could not obtain accurate data on birth weight because at the time of birth of participants no reliable hospital or primary healthcare records were kept on birth, newborn characteristics, maternal profiles or complications during pregnancy in Indonesia. Although recent studies and meta-analyses have shown that low birth weight is an independent predictor for adulthood CVD,^32^ other studies suggest that birth weight is not causal, but rather a surrogate marker of risk for adult CVD.^1^ In this study we obtained data directly from subjects and parental recall. We are confident that the treating midwives, physicians or obstetricians of participants' mothers adopted the standardised and widely accepted definitions of common pregnancy complications. However, we acknowledge the possibility of recall bias, particularly differential recall of past maternal pregnancy complications because of current disease status (ACS or not). In an attempt to avoid this recall bias, during home visits, we directly verified data on pregnancy complications from the cases and controls with the parents, siblings or other closest family members who were present during the pregnancy. Participants and their family members were only informed about the general overall research interest, the possible influence of early life factors on ACS. The pregnancy data pertaining to this study were drawn from an extensive questionnaire, and as this association is not widely considered, it is unlikely that patients or controls and their families have been reporting differentially about the pregnancy complications. Nevertheless, we cannot definitively exclude the possibility of recall bias.

Finally, we could not consistently detect the association between maternal high blood pressure and offspring CHD. The associations were unstable, but the ORs were increased, and therefore this may reflect limited power from our sample size to perform subgroup analysis. Our study was a formal and planned case-control study as apparent from prospective (new) ACS cases collection and incidence density sampling of controls. We did not perform a pre hoc power calculation. Instead, we planned for inclusion of as many cases as possible within a reasonable time frame and managed to include one control for every included case, as described. Notably, ours is one of the biggest case-control studies performed on this subject, certainly in Indonesia. We did attempt to avoid overfitting our models, and therefore we carefully performed stepwise analyses in four adjustment models. Although clear rules of thumb for multivariable (conditional) logistic regression are currently not available,^33^ we did have 10–15 outcome observations (cases) per predictor/variable in our analysis. We believe that our findings do approximate real rate ratios because we only enrolled incident cases (patients with newly diagnosed ACS), and we randomly selected age-matched and sex-matched controls from the same neighbourhoods that the cases came from, at the same time with cases inclusion.

Our findings contribute to the understanding of the relationship between pregnancy complications and the clinically manifest CVD in adult offspring. However, further studies—ideally larger, prospective and longitudinal—are required to confirm the specific associations and to investigate the underlying mechanisms. In terms of clinical implications, optimising maternal health and nutrition during pregnancy may reduce or delay the development of CVD in the offspring. Vaccination and adequate treatment of pregnant women with infectious diseases is of importance, especially in settings where the burden of infection is high. In addition, the necessity of standardised data and management of pregnancy complications is essential, particularly in low-income and middle-income countries in South-East Asia where the infrastructure, financial and human resources are limited.

In conclusion, offspring of mothers with complicated pregnancies are at increased risk of premature ACS in young adulthood. Our finding may be of particular relevance in populations with epidemiological transition, where the incidence of both pregnancy-related morbidity and CVD are high.

**Key messages**

**What is already known about this subject?**

- Maternal complications during pregnancy have long been associated with increased risk factors for cardiovascular disease (CVD) in the offspring. However, it is unclear if these complications are also related to the development of clinically manifest CVD in the offspring, particularly at a younger age.
- Both maternal pregnancy complications and premature CVD are currently major health issues in South-East Asia.

**What does this study add?**

- We found that pregnancy complications were associated with an increased risk of clinically manifest acute coronary syndrome in young adult offspring. This is the first report of an association between the common pregnancy complications (especially maternal infection) and offspring premature coronary heart disease in a low-income and middle-income population of South-East Asia.
- Our study adds to the existing knowledge on maternal pregnancy complications and cardiovascular risk factors, notably that such exposures may partly contribute to the increase of premature CVD in countries with epidemiological transition.

**How might this impact on clinical practice?**

- From our findings, we suggest that optimising maternal health and nutrition during pregnancy may reduce or delay the development of CVD in the offspring.
- Vaccination and adequate treatment of pregnant women with infectious diseases is of importance, especially in settings where the burden of infection is high.
- The necessity of standardised data and management of pregnancy complications is essential, particularly in the low-income and middle-income countries in South-East Asia where the infrastructure, financial and human resources are limited.

## Supplementary Material

[heartasia-2017-010896supp.pdf]
