# Supplementary material for: Pregnancy-related conditions and premature coronary heart disease in adult offspring
Source: Heart Asia. 2017 May 24;9(1):90–5. doi: 10.1136/heartasia-2017-010896 (PMC5730950; doi:10.1136/heartasia-2017-010896)
Supplement: Supplementary data [file heartasia-2017-010896supp001.pdf]

Supplementary file

Methods

We developed a simple questionnaire on dietary pattern by adopting and modifying the National Health and Nutrition Examination Survey (NHANES) food frequency questionnaire. We applied questions on food frequencies and dietary habit corresponding to the local circumstance of Makassar population. We defined high salty food intake as a consumption of salt and MSG in daily food of ≥1 teaspoon/day and/or consumption of local salty/MSG food (i.e. salty fish, bakso, coto, pallubasa, or instant/dried noodle) for ≥3 times/week. High fatty food intake was defined as consumption of local fatty food (i.e. coto, pallubasa, karee, or other traditional meat (beef, buffalo, lamb) dietary) and/or seafood (i.e. crab, prawn, squid, or mussles) for ≥3 times/week. Less fiber was defined as consumption of a serving of fruit, juices, or vegetables for <3 times/week. Furthermore, we adopted physical activity recommendation from the American Heart Association. We defined physical inactivity as never or doing moderate-intensity aerobic activities (i.e. walking, climbing stairs, gardening, yard work, moderate-heavy house work, dancing, or home exercise) for <150 minutes/week, or doing at least 20 minutes of vigorous aerobic exercises (i.e. brisk walking, jogging, swimming, bicycling, or jumping rope) for <3 times/week.[1] For smoking status, we combined the standard National Health Interview Survey (NHIS) and the standard National Survey on Drug Use and Health (NSDUH-S) to differentiate current and former smoker. Current smoker was defined as participant who has smoked at least 100 cigarettes in their lifetime and has smoked in the last 30 days, while former smoker was defined as those who smoked at least 100 cigarettes in their lifetime and has not smoked at all in the last 30 days.[2] A positive family history of CVD was defined as ≥1 first degree and/or ≥2 second degrees family members with CVD before the age of 55 years in men and 65 years in women.[3-5]

Parental history of diabetes mellitus (DM) was positive if participant have mother or father with type 2 DM. Family history of premature sudden cardiac death was positive if ≥1 first-degree and/or ≥2 second-degree family members have died at age <60 years due to CVD.[6] Positive history of hypertension was defined as a known hypertension (systolic blood pressure (BP) ≥140 mmHg and/or diastolic BP ≥90 mmHg[7]), or on medication for hypertension. History of type 2 diabetes mellitus (DM) was defined positive if participant had a known type 2 DM (fasting plasma glucose ≥7.0 mmol/L or 2-hour plasma glucose ≥11.1 mmol/L),[8] or on medication for type 2 DM. Lastly, for current monthly income, we used the cut-off point of ≥Rp.1,810,000,- as the national average of minimum wages for decent living in Indonesia in 2015.[9]

References:

1. Haskell WL, Lee IM, Pate RR, et al. Physical activity and public health: Updated recommendation for adults from the American College of Sports Medicine and the American Heart Association. *Circulation* 2007;116(9):1081-93.

2. Ryan H, Trosclair A, Gfroerer J. Adult current smoking: differences in definitions and prevalence estimates-NHIS and NSDUH, 2008. *J Environ Public Health* 2012;2012:918368.

3. Mulders TA, Meyer Z, van der Donk C, et al. Patients with premature cardiovascular disease and a positive family history for cardiovascular disease are prone to recurrent events. *Int J Cardiol* 2011;153(1):64-7.

4. Lloyd-Jones DM, Nam B, D'Agostino RB, et al. Parental Cardiovascular Disease as a Risk Factor for Cardiovascular Disease in Middle-aged Adults: A Prospective Study of Parents and Offspring. *JAMA* 2004;291(18):2204-11.

5. Nasir K, Budoff MJ, Wong ND, et al. Family history of premature coronary heart disease and coronary artery calcification: Multi-Ethnic Study of Atherosclerosis (MESA). *Circulation* 2007;116(6):619-26.

6. Ranthe MF, Carstensen L, Oyen N, et al. Family history of premature death and risk of early onset cardiovascular disease. *J Am Coll Cardiol* 2012;60(9):814-21.

7. Narkiewwicz K, Redon J, Zanchetti A, et al. ESC Essential Mesages. 2013 ESH/ESC Guidlines for the management of arterial hypertension. *Eur Heart J* 2013;34:2159-219.

8. World Health Organization-International Diabetes Federation. Definition and diagnosis of diabetes mellitus and intermediate hyperglycemia : report of a WHO/IDF consultation. Geneva, Switzerland,: 2006.

9. Pemerintah.net. Daftar Lengkap UMP 2015. http://pemerintah.net/daftar-lengkap-ump-2015/ (accessed 02 February 2015).
